# IL-32 as a potential biomarker and therapeutic target in skin inflammation

**DOI:** 10.3389/fimmu.2023.1264236

**Published:** 2023-09-01

**Authors:** Alexandra Wallimann, Mirjam Schenk

**Affiliations:** ^1^ Christine Kühne – Center for Allergy Research and Education (CK-CARE), Davos, Switzerland; ^2^ Institute of Tissue Medicine and Pathology, Experimental Pathology, University of Bern, Bern, Switzerland

**Keywords:** IL-32, atopic dermatitis, skin inflammation, melanoma, infection, CTCL

## Abstract

IL-32 is a recently described cytokine that performs a variety of functions under inflammatory conditions. Serum IL-32 has been shown to be elevated in several diseases, including type 2 diabetes, cancer, systemic lupus erythematosus, HIV infection, and atopic diseases including atopic dermatitis. There are nine different isoforms of IL-32, with IL-32γ being the most biologically active one. The following review summarizes the different roles of the various IL-32 isoforms in the context of skin inflammation, with a focus on atopic dermatitis.

## Take-home messages

- IL-32 levels in skin lesions of atopic dermatitis, hidradenitis suppurativa and alopecia areata are increased compared to healthy skin.
- In atopic dermatitis, IL-32 plays a key role in the interaction between keratinocytes and Langerhans cells and contributes to the inflammatory process, providing a potential therapeutic target.
- Intratumoral IL-32γ promotes an inflammatory tumor microenvironment in melanoma, which is associated with prolonged overall survival and better response to current therapies.
- IL-32 supports T cell mediated host defence in microbial skin infections.

## Introduction

Inflammatory skin disorders comprise a wide range of conditions such as atopic dermatitis (AD), psoriasis, hidradenitis suppurativa (HS), alopecia areata (AA), and others. AD is the most prevalent inflammatory skin disease affecting up to 20% of children and 10% of adults (1). AD is characterized by red eczematous patches on the skin and repetitive itch-scratch cycles, leading to a reduced quality of life and an increased risk of developing other atopic diseases such as food allergies, allergic rhinitis, or asthma (2). In addition to an increased risk of developing atopic comorbidities, AD has also been shown to be associated with cardiovascular diseases and mental health comorbidities, including anxiety, depression and attention-deficit hyperactivity disorder (ADHD) (3). Although AD is primarily a childhood disease, it can persist throughout a person’s lifetime and flare up during adulthood. Both, genetic factors, such as Filaggrin mutations, and environmental factors, like urban or rural living environment, pollution, and nutrition, are involved in disease induction and progression. While the basic principles of immune activation in AD are understood, it remains unclear why the disease course is so heterogenous, varying from complete remission to recurrent flares. Several treatment strategies are currently available, including local (topical) treatment with e.g. corticosteroids or calcineurin inhibitors, which can be applied reactively or proactively, systemic treatment with classical immunosuppressants such as cyclosporin, methotrexate or corticosteroids, JAK-inhibitors, or biologicals such as dupilumab and tralokinumab (4). However, treatment responses vary significantly between patients, indicating the existence of different disease endotypes. This underscores the need for a personalized medicine approach to successfully treat AD patients. Novel biomarkers, including cytokines such as IL-32, could become an essential tool for stratifying patients according to disease severity, predicting disease course, and treatment response.

Generally, disruption of the epidermal barrier is considered the origin of AD pathophysiology (5). This disruption can be caused and influenced by genetic mutations of epidermal barrier components, such as the loss-of-function mutation in filaggrin, a dysbalanced skin microbiome, such as a high abundance of *Staphylococcus aureus* leading to a dysbiosis, and other environmental factors. Consequently, epidermal-derived cytokines, also known as alarmins, including TSLP, IL-33, and IL-25 are secreted, initiating the innate skin-derived immune response. Due to the leaky barrier, microbial and environmental antigens can easily penetrate the epidermis and influence the local immune response. Upon antigen presentation, naïve T cells are primed towards effector Th2 cells. The local immune activation in the skin further develops into systemic immune activation, resulting in a chronic, low-grade inflammatory state and IgE-mediated sensitization. The initial innate immune response plays a pivotal role in the onset of the disease and the progression of local to systemic inflammation. IL-32 is potentially one of the key cytokines in shaping immune activation and inflammation in the early stages of AD development, as it has the capacity to strongly influence dendritic cell (DC) activation (6). Lesional skin of AD patients was found to have increased levels of IL-32 (7). In addition, IL-32 serum levels of AD patients were shown to be increased and correlated with disease severity (7, 8). Treatment with oral cyclosporin for 28 days was found to decrease IL-32 levels in the serum of AD patients, which was accompanied by an alleviation of skin lesions in these patients (9). Besides its role in AD, IL-32 has also been proposed as a biomarker for psoriasis, AA and HS. Therefore, this review focuses on the biological functions of IL-32, particularly in AD and other inflammatory skin disorders (**Table 1**), and evaluates its potential as a biomarker and therapeutic target in these diseases.

**Table 1:** Overview of current publications showing roles of IL-32 in skin inflammation.

Year	Authors and Journal	Main findings
Atopic Dermatitis		
**2010**	Meyer et al. (7) *Journal of Allergy and Clinical Immunology*	IL-32 was produced and released by KCs in AD
**2013**	Choi et al. (10) *Food and Chemical Toxicology*	IL-32 was reduced in lesional skin upon Galangin (flavonoid) treatment
**2014**	Kim et al. (11) *Experimental Biology and Medicine*	IL-32 was reduced in lesional skin upon Rutin (flavonoid) treatment
**2017**	Kitayama et al. (9) *Journal of the European Academy of Dermatology and Venereology*	IL-32 in serum of AD patients was reduced upon 28 days cyclosporin treatment and associated with alleviation of AD symptoms
**2020**	Lee et al. (12) *Journal of Allergy and Clinical Immunology*	IL-32γ transgenic mice showed a reduced inflammatory AD response
**2022**	Chang et al. (8) *Journal of Translational Medicine*	IL-32 was higher expressed in skin and blood samples in AD patients compared to healthy controls and correlated with AD disease severity
Psoriasis		
**2010**	Kempuraj et al. (13) *European Journal of Dermatology*	*IL32* gene expression was increased in psoriatic skin lesions *IL32* was induced in LAD2 human mast cells upon stimulation with substance P
Hidradenitis suppurativa		
**2017**	Thomi et al. (14) *British Journal of Dermatology*	IL-32 expression was higher in serum and skin lesions of HS compared to psoriasis, AD and healthy subjects
Alopecia areata		
**2016**	Fuentes-Duculan et al. (15) *Experimental Dermatology*	IL-32 was expressed in AA skin lesions Methylprednisolone treatment reduced IL-32 in AA skin lesions and was associated with hair regrowth
Skin cancer		
**2016**	J. Lee et al. (16) *Oncotarget*	IL-32α-overexpressing human melanoma cells showed increased migratory ability *in vitro*
**2016**	Nicholl et al. (17) *Journal of Surgical Oncology*	IL-32α administration inhibited HTB-72 melanoma cell proliferation *in vitro*
**2018**	Y.S. Lee et al. (18) *Journal of Experimental & Clinical Cancer Research*	IL-32γ transgenic mice were shown to have reduced stemness and a lower inflammatory tumor microenvironment compared to WT mice
**2020**	Gruber et al.(19) *JCI Insight*	IL-32γ was associated with activated myeloid cells, increased overall survival, and correlated with a T cell-inflamed tumor microenvironment in melanoma
**2021**	Kang & Kim (20) *Journal of Clinical Medicine*	*IL32 mRNA* expression in cutaneous melanoma correlated with NK cell infiltration and the presence of cytolytic granzyme and perforin, which was associated with a good prognosis in cutaneous melanoma patients
CTCL		
**2012**	Van Kester et al. (21) *Journal of Investigative Dermatology*	*IL32* mRNA was highly expressed by malignant T cells in MF Serum IL-32 levels correlated with disease activity in MF patients
**2014**	Suga et al. (22) *Journal of Investigative Dermatology*	IL-32 was expressed by KCs in lesional skin of MF patches IL-32 was expressed by dermal T cells in MF tumors
**2017**	Ohmatsu et al. (23) *OncoImmunology*	IL-32 in MF skin lesions promoted DC differentiation of monocytes to a CD1c^+^ skin resident phenotype
**2022**	Yu et al. (24) *Journal of Investigative Dermatology*	IL-32 promoted the survival of malignant T Cells by inducing and maintaining a CD14^+^ APC population
Skin infection		
**2011**	Zepp et al. (25) *Journal of Immunology*	IL-32 was shown to have a direct antiviral effect on HSV DNA replication and ability to reduce viral load
**2012**	Peng et al. (26) *Journal of Virology*	*IL32* was expressed at the dermal-epidermal junction of human genital skin biopsies and showed an anti-viral function in herpes simplex virus infection
**2012**	Schenk et al. (27) *Nature medicine*	IL-32 induced differentiation of monocytes into CD1b^+^ DCs, which were more potent at antigen (cross-)presentation to CD8^+^ T cells, thereby promoting an adaptive immune response in mycobacterial infections
**2014**	Galdino et al. (28) *BMC Infectious Diseases*	IL-32 expression in human cutaneous lesions was induced by *Leishmania amazonensis*
**2017**	Gomes et al. (29) *Parasites and Vectors*	IL-32γ supported the healing of infection-associated skin lesions by enhancing the immune response against *L. amazonensis* IL-32γ promoted skin healing in *Leishmania braziliensis* infection
**2018**	Gonnet et al. (6) *British Journal of Dermatology*	IL-32 was produced in an *ex vivo* human skin explant model in presence of MVA infection-induced KC death

## Biological properties of IL-32

IL-32 is a cytokine initially cloned from human natural killer (NK) cells and its original name was therefore natural killer cell transcript 4 (NK-4) (30). IL-32 is encoded by nine different isoforms through alternative splicing (IL-32α, IL-32β, IL-32γ, IL-32δ, IL-32θ, IL-32ε, IL-32ζ, IL-32η, and IL-32s) (31). The three most extensively studied isoforms are IL-32α, IL-32β and IL-32γ, with IL-32β being the most abundant and IL-32γ being the longest and most biologically active (32, 33). IL-32 induces the production of various cytokines such as TNF-α, IL-8, IL-1β and MIP-2, and exhibits little homology with other cytokines (30). Although the murine orthologue of IL-32 remains unidentified, studies have demonstrated that stimulation of murine macrophages and DCs with human IL-32 leads to activation of similar signalling cascades and biological processes, indicating the existence of the IL-32 receptor and pathway in rodents (19, 27, 30). The role of IL-32 *in vivo* has been investigated using IL-32 transgenic mice (34–36) and bone marrow transplantation of retroviral constructs containing human IL-32β (37), or application of recombinant human IL-32γ (12, 19).Various immune and non-immune cells including T lymphocytes, NK cells, monocytes/macrophages, DC, and epithelial cells (e.g. keratinocytes) have been shown to express IL-32 (31). A comprehensive comparison showed that lymphocytes and NK cells express the highest levels of *IL32*, and that IL-32 protein expression was highest in activated effector T cells (19). The presence of a microbial infection, oxidative stress, and pro-inflammatory cytokines (e.g. TNF-α or IL-12) can substantially increase IL-32 expression (31). Depending on the triggering factors, the isoform, and cell type, IL-32 mediates its effect primarily intracellularly, but release of IL-32 by extracellular vesicles of myeloma cells has been described (38). Synovial fibroblasts in rheumatoid arthritis have also been shown to secrete low amounts of IL-32 (39). Furthermore, IL-32 measured in serum or cell culture supernatant suggests the presence of released, extracellular IL-32. To date, the cell surface receptor responsible for binding extracellular IL-32 has not yet been identified. However, extracellular IL-32γ triggers the MAPK/ERK pathway in myeloid cells, thereby inducing their maturation, activation, and chemokine secretion (19). The binding of IL-32α and IL-32β to integrin extracellular domains activates pro-caspase-3 signalling and thus leads to cell death (40). It has also been shown that IL-32 can induce apoptosis in T cells and keratinocytes (KCs) (7, 32).

Furthermore, IL-32 can induce differentiation, maturation and cross-priming in DCs as well as M1 polarization and cytokine/chemokine secretion in macrophages (19, 27). IL-32 can amplify inflammatory cytokines induced by microbial receptors, such as Toll-like receptors (TLR) and intracellular nuclear oligomerization domain (NOD) receptor families and can trigger TLR signalling in the absence of PRR ligands through the protease-activated receptor 2 (PAR2) (41, 42).

Although IL-32 is generally considered pro-inflammatory due to its ability to activate NFκB signalling and p38 MAPKs and enhance tumor immunity, it can exert anti-inflammatory effects under certain circumstances (19, 27, 40). This will be discussed in more detail in the upcoming section, with a focus on skin inflammation. It is noteworthy that the major isoforms of IL-32, namely IL-32α, IL-32β, IL-32γ, and IL-32δ have been detected in both skin and blood samples from individuals with AD, psoriasis, and HS (7, 14).

## The role of IL-32 in healthy skin and atopic dermatitis

IL-32γ has been described as key molecular link between KCs and Langerhans cells (LC) under steady-state conditions in healthy skin, where it induces the migration of LC from the epidermis into the dermis before they migrate to the draining lymph node (6). LC and other DC subsets in the skin were shown to express IL-32. The expression of the four main IL-32 isoforms (α, β, γ and δ), particularly IL-32α, was found to be higher in epidermal LCs and dermal CD1a^dim^CD141^-^ DCs compared to dermal CD1a^dim^CD141^+^ DCs and CD14^+^ DCs. Furthermore, IL-15 was shown to induce IL-32α expression in these DC subsets. While IL-15 is inducing cytolytic capacity of NK cells, DC-derived IL-32α is antagonizing the IL-15 mediated NK cell effector molecule expression and killing capacity, suggesting a feedback mechanism of IL-32α (43). Importantly, based on expression data of the human protein atlas, T cells appear to be the largest producer of IL-32 in healthy skin (44).

**Figure 1:**
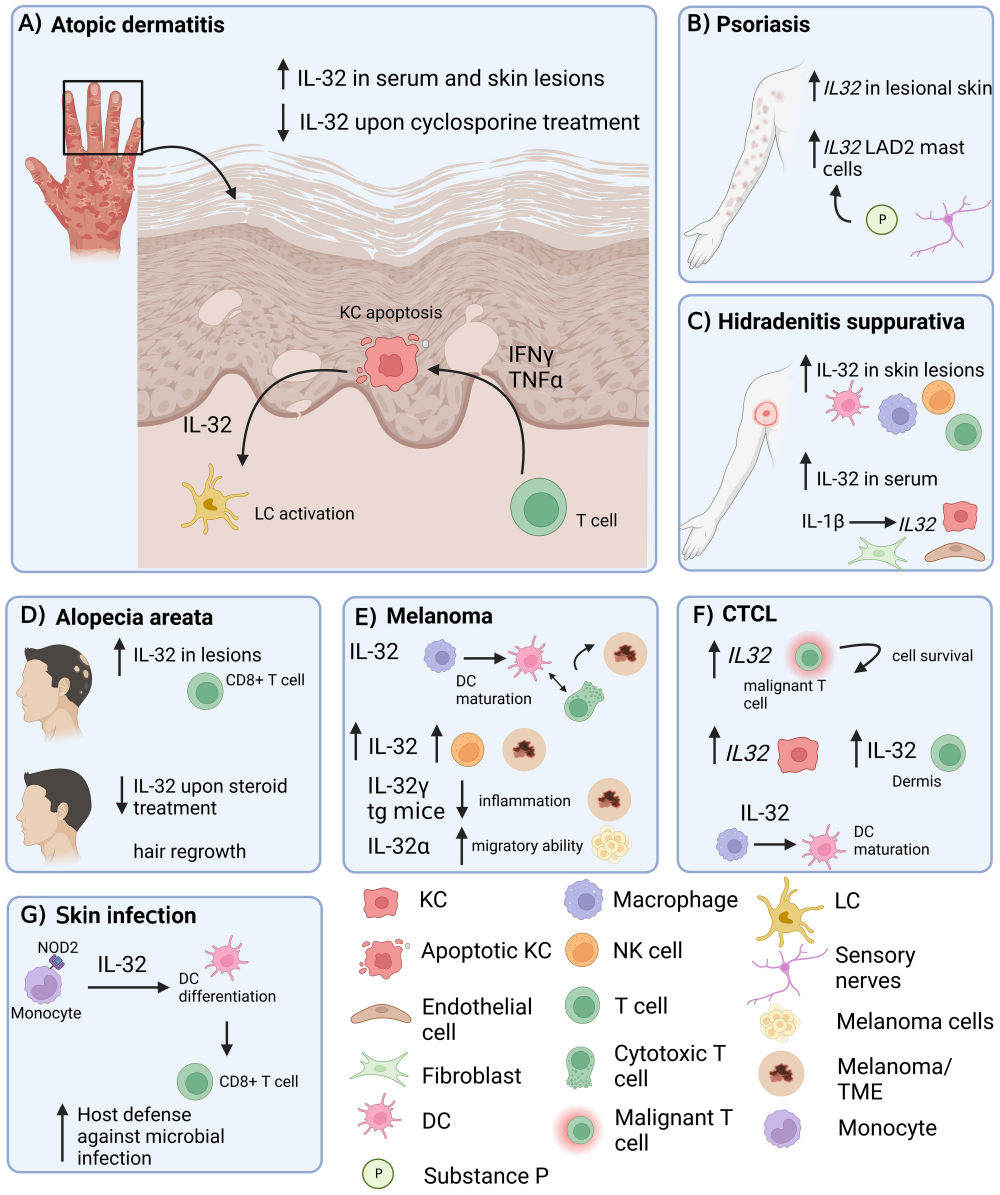
Role of IL-32 in different inflammatory skin diseases. IL-32 has been shown to be expressed in skin lesions in various inflammatory conditions. **(A)** AD: IL-32 produced by KCs is dependent on T cell-derived IFNγ and TNF-α and drives LC activation. **(B)** Psoriasis: *IL32* gene expression is increased in lesional psoriatic skin. **(C)** HS: IL-32 is increased in lesional HS skin and in serum. IL-1β induces *IL32* expression in KCs, fibroblasts and endothelial cells. **(D)** IL-32 is elevated in lesional AA skin, produced by CD8^+^ T cells and decreased upon intra-lesional methylprednisolone treatment. **(E)** Melanoma: IL-32 is driving DC maturation and supporting a T cell inflamed tumor microenvironment in melanoma. **(F)** In CTCL, *IL32* was expressed by malignant T cells and KCs. IL-32 was driving DC maturation. **(G)** Skin Infection: IL-32 was driving DC differentiation and antigen cross-presentation to favour T cell mediated host defence in microbial infections. AD, atopic dermatitis; KC, keratinocyte; LC, Langerhans cells; AA, alopecia areata; HS, Hidradenitis suppurativa; DC, dendritic cell; NK cell, natural killer cell; LAD2, laboratory of allergic diseases 2 (human mast cells cell line); CTCL, cutaneous T cell lymphoma; TME, Tumor microenvironment; tg, transgenic. Figure created in biorender.com.

Evidently, IL-32 is upregulated in the skin and serum of AD patients, and serum levels of IL-32 correlate with disease severity, even in patients who respond to treatment. Although IL32 was first identified in activated T and NK cells (45), current literature suggests that KC appear to be an important source of IL-32 in skin inflammation, particularly in AD (6, 7). In addition, IL-32 has been reported to induce apoptosis of KCs. Expression of IL-32 is upregulated in KCs in response to various inflammatory stimuli, such as proinflammatory cytokines, and microbial components (7). Although IL-32 is known to be produced by KCs, it is not actively secreted by them, instead, KC death is required to release IL-32 (**Figure 1A**). This released IL-32 can promote the activation and migration of immune cells, such as T cells and neutrophils, which further contribute to the inflammatory response in the skin. Since it has been shown that in viral infections of the skin (MVA), KC-released IL-32 can activate LC (6), IL-32 may also play an important role in the activation of LC and the priming of T cells in AD.

Another study showed that IL-32 promotes AD through JAK-miRNA-155 signalling (8). In this study, the expression of IL-32 in skin and blood samples was higher in AD than in healthy control samples, both in an IL-32 transgenic murine AD model induced by phthalic anhydride (PA) and in human patients. In a DFE/DNCB induced murine dermatitis model, two flavonoids, Galangin and Rutin, were found to suppress IL-32 in lesional skin of these mice, which was associated with reduced clinical scores and lymphocyte proliferation, as well as lower serum IgE levels, highlighting the importance of IL-32 in inflammatory skin lesions (10, 11).

While many studies have emphasized the pro-inflammatory properties of IL-32 and its role in AD progression, Sun Lee et al. found that IL-32γ transgenic mice showed reduced inflammation in PA and MC903 AD models. The reduced manifestations of AD symptoms in IL-32γ transgenic mice was likely a consequence of a lower inflammation in these mice compared to the WT mice, characterized by reduced infiltration of pro-inflammatory immune cells, mast cells, and downregulated Th1, Th2 and Th17 associated cytokines. In contrast, IL-32γ transgenic AD mice showed higher numbers of Foxp3^+^ Treg cells than WT AD mice (12). These discordant findings could be due to the use of IL-32γ transgenic mice, which could develop an immunosuppressive response due to the continuous expression of IL-32γ. This will be further elaborated in the discussion section of this review.

In summary, IL-32 has pro-inflammatory capabilities and plays a role in the interaction between KCs and LCs in human AD skin. While T cells have been shown to be the main source of IL-32 in other inflammatory conditions, KC-produced IL-32 seems to play and important role in AD. Further studies will elucidate the complex interplay between the cellular sources and targets of IL-32 in AD.

## The role of IL-32 in other inflammatory skin conditions: Psoriasis, hidradenitis suppurativa and alopecia areata

In addition to its role in AD, IL-32 has also been shown to be involved in other inflammatory skin diseases such as psoriasis (**Figure 1B**), HS (**Figure 1C**) and AA (**Figure 1D**). Psoriasis is a chronic inflammatory skin disease which is appearing either in separated pustular forms or in chronic plaque forms. IL-17 and IL-23 are key drivers in the pathogenesis of psoriasis and indicate a Th17-dominated inflammation (46). One study showed increased *IL32* gene expression in psoriasis lesions compared to healthy controls (13) (**Figure 1B**). In addition, expression of the *IL32* gene in human mast cells (LAD2) was induced upon stimulation with substance P, a mediator released in the skin from sensory nerve endings, indicating potential induction of IL-32 during a neurogenic flare in psoriasis (13). However, IL-32 protein was not detected in the skin of psoriasis patients, only in AD and HS patients (7, 14). A key difference between AD and psoriasis is that KC in psoriasis tend to proliferate rather than undergo apoptosis (47). Since KC apoptosis appears to be required for the release of IL-32 in AD, this would explain the low abundance of IL-32 protein in psoriasis and imply that KC-derived IL-32 may not be involved in the pathogenesis of psoriasis.

Also characterized as a chronic inflammatory skin disease, HS presents with deep-seated nodules, mainly located in the axilla, inguinal and perianal area and sub-mammary folds, that further expand to abscesses (48). Thomi et al. showed that IL-32 protein expression was higher in HS skin lesions as well as in serum compared to skin from AD and psoriasis lesions. Natural killer cells, T cells, macrophages and DCs were found to be the producers of IL-32 in HS skin lesions (**Figure 1C**). In addition to elevated IL-32 protein levels, total *IL32* gene expression was also higher in HS skin. The mRNA expression levels of *IL32* positively correlated with the degree of inflammation in HS skin lesions. Although *IL32* correlated positively with the numbers of T cells and macrophages, as well as with IFN-γ and IL-17, no correlation was observed with IL-13, an important Th2 cytokine in AD pathogenesis (14). In another study, a highly active IL-1β pathway was detected in HS skin lesions compared to healthy and psoriatic skin, and in a skin explant model, IL-1β was shown to induce *IL32* gene expression in endothelial cells, fibroblasts, and KCs (49) (**Figure 1C**).

AA is an autoimmune inflammatory skin disease characterized by T cell infiltration targeting hair follicles and thus causing patchy hair loss mainly on the scalp (50). It was shown that IL-32 is increased in AA scalp lesions compared to non-lesional regions, and that CD3^+^ and CD8^+^ T cells are expressing IL-32. Upon intralesional methylprednisolone treatment, IL-32 expression was decreased and associated with hair regrowth in these regions (**Figure 1D**). Thus, IL-32 was proposed to be a potential biomarker for disease pathogenesis and hair loss in AA (15). The precise mechanism of how IL-32 promotes the pathogenesis of AA is currently unknown and needs further investigation. Furthermore, it would be interesting to see if blocking IL-32 is sufficient to reduce inflammation and induce hair regrowth.

In summary, IL-32 protein expression seems to be increased in HS and AA lesions compared to healthy skin, whereas only *IL32* gene expression is increased in psoriasis. Depending on the inflammatory skin condition, IL-32 appears to originate from different cell types including T cells, KCs, DCs, endothelial cells, and fibroblasts. The use of single cell technologies will help to identify the precise cellular sources and targets of IL-32 in each condition.

## IL-32 in skin cancer and cutaneous T-cell lymphoma

Skin cancer is the most common type of cancer worldwide, and its incidence has steadily increased in recent decades. Ultraviolet (UV) exposure is the most important risk factor causing skin cancer by inducing genetic mutations. Three main types of skin cancer exist including basal cell carcinoma, squamous cell carcinoma, and malignant melanoma (51). Different studies have found that IL-32 plays a role in both, the development, and the induction of an immune response in melanoma (**Figure 1E**) and cutaneous T-cell lymphoma (CTCL, **Figure 1F**).

*IL32* mRNA expression in cutaneous melanoma was correlated with the infiltration of NK cells and the presence of cytolytic granzyme and perforin, which was associated with a good prognosis in cutaneous melanoma patients (20). Another study showed that IL-32γ transgenic mice had reduced carcinogen-induced tumor incidence compared to WT mice, which was associated with a lower inflammatory tumor microenvironment (TME) compared to WT mice. IL-32γ transgenic mice showed reduced gene expression of a variety of pro-inflammatory cytokines including TNF-α, Il-1β, IL-6, as well as Th2-associated IL-4 and IL-13, but increased expression of IL-10 in the skin (18). Eventually, this study showed the ability of IL-32γ to suppress skin carcinogenesis by dampening inflammation within the TME during cancer development. However, Gruber et al. showed that IL-32γ was associated with activated myeloid cells, increased overall survival, and correlated with a T cell-inflamed TME in human melanoma (19). In a murine melanoma model, intratumoral treatment with IL-32γ induced maturation of cross-presenting DC and M1 macrophage polarization. The induced DCs and M1 macrophages act in concert to prime and recruit tumor-specific CD8^+^ T cells into the TME, leading to an increased number of tumor-specific cytotoxic CD8^+^ T cells. Therefore, intratumoral IL-32γ is promoting an inflammatory TME, which is associated with increased overall survival and better cancer diagnosis. Intratumoral IL-32γ could function as a novel potent adjuvant in immunotherapy to enhance the efficacy of immune checkpoint blockade.

The effect of IL-32 on melanoma cells has also been studied. While administration of recombinant IL-32γ did not alter the transcriptome of melanoma cells (19) treatment of a human melanoma cell line (HTB-72) with recombinant IL-32α resulted in decreased proliferation of these cells, due to increased expression of the anti-proliferative p21 and p53 in these cells (17). In contrast, IL-32α-overexpressing human melanoma cells showed increased migratory ability *in vitro* caused by reduced E-cadherin expression favouring F-actin polymerization. The increased migratory capability of IL-32α overexpressing melanoma cells was associated with higher lung metastasis *in vivo* (16). This discrepancy could be due to the different signalling pathways triggered by intracellularly expressed versus extracellularly administered IL-32α.

CTCL is a type of non-Hodgkin’s lymphoma that affects the skin. It occurs when a mutation or abnormality develops in T-cells, causing them to multiply uncontrollably and accumulate in the skin. The exact cause of CTCL is not fully understood, but it is thought to be related to a combination of genetic and environmental factors. Mycosis fungoides (MF) is the most common type of CTCL, accounting for about 50% of all cases. MF typically starts as a red, scaly rash on the skin, which can be mistaken for other skin conditions such as eczema or psoriasis. Over time, the rash can develop into plaques or tumors and spread to other parts of the body, such as the lymph nodes, blood, and internal organs. The malignant T cells in MF express high and consistent *IL32* mRNA and serum IL-32 levels correlated with disease activity in MF patients (21). Consistent with this study, Suga et al. found that *IL32* mRNA was higher in lesional skin of MF patches compared to normal skin. KCs were the main producers of IL-32 in the lesional skin of MF patch and plaque, while T cells in the dermis expressed IL-32 in MF tumors (22). Thus, IL-32 might play a role in the formation and persistence of CTCL lesions and could be a potential therapeutic target. On the other hand, IL-32β and IL-32γ have been shown to accelerate the induction of CD1c^+^ mDCs and CD163^+^CD68^+^ macrophages expressing IDO in MF skin lesions and to contribute to a tolerogenic tumor environment (23). In addition, IL-32 was found to be the most abundantly expressed cytokine in MF. Single-cell sequencing revealed that *IL32* was mainly expressed by malignant and regulatory T cells, but also by a subset of KCs. Recombinant IL-32β did not affect malignant T cell survival *in vitro.* However, in the presence of myeloid cells, IL-32β induced and maintained a CD14^+^ APC population, which was associated with enhanced T cell survival. Thus, IL-32β produced by malignant T cells favours a protective niche for T cell survival in CTCL (24).

## IL-32 in skin infections

Amongst its key role in different chronic inflammatory skin conditions, there is evidence that IL-32 is also involved in host defense against infectious pathogens (**Figure 1G**). IL-32 has been shown to be associated with the control and immunopathology of numerous infectious diseases including tuberculosis, HIV, leprosy, and hepatitis.

In the context of skin infections, IL-32 has been shown to play a supportive role in the healing of infection-related skin lesions (29). Infection with *Leishmania amazonensis* induced high IL-32 expression which enhanced the immune response against the pathogen and supported healing of skin lesions (28, 29). Similarly, IL-32γ promoted skin healing of skin lesions associated with *Leishmania braziliensis* infection, indicating its crucial role in controlling parasite load and dissemination (29).

In leprosy, a human mycobacterial infection, IL-32 has been shown to promote the differentiation of monocytes into CD1b^+^ DCs upon NOD2 activation. These CD1b^+^ DCs were more efficient in antigen cross-presentation to CD8^+^ T cells, thereby promoting an adaptive immune response. Accordingly, the expression of NOD2 and IL-32, as well as the number of CD1b^+^ DCs were higher in tuberculoid than in lepromatous leprosy lesions, indicating a relevant mechanism to control the dissemination of infection (27).

Regarding viral infections, an antiviral function of IL-32 has been demonstrated in herpes simplex virus (HSV) infection, where CD8^+^ T cells expressed *IL32* at the dermal-epidermal junction of human genital skin biopsies (26). IL-32 has previously been shown to have a direct antiviral effect on HSV DNA replication and to reduce viral load (25). In an *ex vivo* human skin explant model, modified vaccinia virus Ankara (MVA) infection induced KC death, accompanied by production of IL-32 (6). IL-32 induced morphological changes in LCs by shortening dendrites, downregulating adhesion molecules, and inducing release of CXCL10, while upregulating CD80 and HLA-DR, which are hallmarks of LC maturation and migration. Blocking IL-32 with siRNA and an anti-IL-32 antibody significantly inhibited LC activation. Several TLR ligands, including those for TLR-1, 2, 4, and 7 induced IL-32 production by KCs, which subsequently initiated LC activation. This is consistent with the finding that deficiency of MyD88, a universal adaptor molecule for TLRs, in KCs directly impaired LC migration upon antigen exposure (52).

In summary, IL-32 has been shown to support the clearance of viral and bacterial pathogens in the skin, which in turn has been associated with healing of microbial-related skin lesions.

## Discussion

In recent years, IL-32 has emerged as a novel pro-inflammatory cytokine that plays a central role in various inflammatory conditions such as rheumatoid arthritis and inflammatory bowel disease, but also in infections and cancer. This review highlights that IL-32, particularly IL-32α, β, and γ, also play a pro-inflammatory role in the pathogenesis of various inflammatory skin disorders. Several studies have shown an association between IL-32 levels and degrees of inflammation in AD, psoriasis, HS, and AA.

Remarkably, studies using IL-32 transgenic mice or IL-32 overexpressing cells *in vitro* often report an anti-inflammatory effect of IL-32 in various disease models. In AD, IL-32γ transgenic mice showed reduced inflammation compared to WT mice (12). Similarly, IL-32β overexpressing mice showed reduced levels of proinflammatory cytokines TNF-α, IL-6, and IL-1β in a murine model of rheumatoid arthritis, which improved arthritic inflammation in these mice (35). However, exacerbation of collagen-induced arthritis was observed in mice given human IL-32β by bone marrow transplantation (37). Also, application of recombinant IL-32γ in mice resulted in an increase in inflammatory cell infiltration and tissue damage in arthritic mice (53). Furthermore, in the dextran-sodium-sulfate (DSS)-induced colitis model, IL-32γ transgenic mice initially showed a more severe disease course than WT mice. However, after 6 days, the colitis regressed, which was accompanied by an increase in colonic IL-10 levels. (36). Together, these findings indicate that models in which IL-32 is continuously expressed are more likely to elicit immunosuppressive responses in the long-term, which may be a physiological response to the continuous action of IL-32. The use of IL-32 transgenic mice or overexpressing cell lines needs to be carefully evaluated and compared to exogenous administration or bone marrow transplantation of IL-32 to fully elucidate its role.

While KCs have been identified as the major cellular source of IL-32 in AD in an *in vitro* model with limited cell types (7), a recent paper has shown that *IL32* is expressed by COL18A1^+^ fibroblasts in AD skin (54). Further studies using single cell analysis and spatial resolution will elucidate the precise cellular source and the role of IL-32 during the course of the disease. Inhibition of IL-32 or its deactivation could be a potential approach to reduce skin inflammation. As IL-32 is activated by proteinase-3, Marcondes et al. used the serine protease inhibitor α-1 antitrypsin to suppress IL-32, although α-1 antitrypsin is not a specific inhibitor to IL-32 (55). The suppression of IL-32 by α-1 antitrypsin was associated with reduced T cell proliferation in an allogeneic murine marrow transplantation model. Studies using IL-32 knockdown in human cell lines may also shed light on whether IL-32 can be used as a potential therapeutical target to reduce inflammation.

In general, IL-32 is an interesting target to treat skin inflammation. However, the development of specific IL-32 inhibitors is hampered by the fact that IL-32 can act both intracellularly and extracellularly, that a specific extracellular IL-32 receptor is still elusive, and that the murine orthologue of IL-32 remains unidentified. In addition, the role of the different IL-32 isoforms in skin inflammation remains to be fully elucidated.

In conclusion, IL-32 is elevated locally and systemically in various inflammatory skin disorders and decreases with anti-inflammatory therapies in AD and AA, leading to improvement in disease symptoms. IL-32 could therefore be considered as a novel potential therapeutic target, particularly in AD and AA, to reduce skin inflammation.

## Author contributions

AW: Conceptualization, Visualization, Writing – original draft. MS: Conceptualization, Funding acquisition, Supervision, Writing – review & editing.

## Glossary

**Table:**

AA	Alopecia areata
AD	Atopic dermatitis
ADHD	Attention-deficit hyperactivity disorder
APC	Antigen presenting cell
CXCL	C-X-C motif ligand
CD	Clusters of differentiation
CTCL	Cutaneous T cell lymphoma
DC	Dendritic cell
DFE	*Dermatophagoides farinae* extract
DNCB	Dinitrochlorobenzene
ERK/MAPK	Extracellular signal-regulated kinase
HIV	Human immunodeficiency virus
HSV	Herpes simplex virus
HS	Hidradenitis suppurativa
IL	Interleukin
ILC	Innate lymphoid cell
JAK	Janus kinase
KC	Keratinocyte
LC	Langerhans cell
MAPK	Mitogen-activated protein kinase
MF	Mycosis fungoides
MIP	Macrophage inflammatory protein
MVA	Modified vaccinia virus Ankara
NFκB	Nuclear factor kappa light chain enhancer of activated B cells
NK	Natural killer (cell)
NOD	Nucleotide oligomerization domain
PA	Phthalic anhydride
PAR	Protease-activated receptor
PRR	Pattern recognition receptor
Th	T helper (cell)
TLR	Toll-like receptor
TME	Tumor microenvironment
TNF	Tumor necrosis factor
TSLP	Thymic stromal lymphopoietin
WT	Wildtype
